# Presenting and exploring biological pathways with PathVisio

**DOI:** 10.1186/1471-2105-9-399

**Published:** 2008-09-25

**Authors:** Martijn P van Iersel, Thomas Kelder, Alexander R Pico, Kristina Hanspers, Susan Coort, Bruce R Conklin, Chris Evelo

**Affiliations:** 1 Department of Bioinformatics, BiGCaT Maastricht University, Universiteitssingel 40, 6229 ER Maastricht, the Netherlands; 2 Gladstone Institute of Cardiovascular Disease, 1650 Owens Street, San Francisco, CA 94158, USA; 3 Departments of Medicine, and Molecular and Cellular Pharmacology, University of California, San Francisco, CA 94158, USA

## Abstract

**Background:**

Biological pathways are a useful abstraction of biological concepts, and software tools to deal with pathway diagrams can help biological research. PathVisio is a new visualization tool for biological pathways that mimics the popular GenMAPP tool with a completely new Java implementation that allows better integration with other open source projects. The GenMAPP MAPP file format is replaced by GPML, a new XML file format that provides seamless exchange of graphical pathway information among multiple programs.

**Results:**

PathVisio can be combined with other bioinformatics tools to open up three possible uses: visual compilation of biological knowledge, interpretation of high-throughput expression datasets, and computational augmentation of pathways with interaction information. PathVisio is open source software and available at .

**Conclusion:**

PathVisio is a graphical editor for biological pathways, with flexibility and ease of use as primary goals.

## Background

The concerted actions of genes, proteins, and metabolites are often conceptualized as pathway diagrams. Pathways represent a familiar concept in biological research, and software designed to work with pathways can help the researcher to organize information related to research questions. Here we present PathVisio, a visualization tool for managing biological pathways, and show several ways in which this tool can facilitate the process of doing research.

It is often said that an image is worth a thousand words, and this is especially true for describing complex interactions among biomolecules. Pathways are commonly used with great effect as teaching aides in textbooks, as notes in lab journals, and as explanatory slides in research presentations. However, a pathway that is drawn in a notebook or presentation software is just a static image. The usefulness of a pathway could be increased dramatically if the software knows something about the biological entities it represents. For example, one could click on entities of a pathway to view the Ensembl page of a relevant gene in a browser window. Ideally, textbook pathways could be combined or compared with other versions of the pathway and stored in an online repository. New pathway information could be compiled from the latest experiments and discoveries. Large experimental datasets would be more understandable through pathways. Clearly, user-friendly software would be helpful for dealing with biological pathways.

A new program, PathVisio, is based on many design principles derived from GenMAPP[1], a popular software suite among bench biologists (which includes MAPPFinder [2] for finding biologically relevant pathways). PathVisio is designed to augment GenMAPP, replacing some but not all aspects of the software by using more flexible technologies such as XML and Java that allow for completely new features and increase the possibilities for future extension. PathVisio is suitable for the creation and exploration of pathways, while relying on GenMAPP for visualization of experimental data and MAPPFinder for statistical analysis.

PathVisio improves GenMAPP in four separate areas. First, PathVisio is written in the Java programming language as opposed to Visual Basic. Thus, PathVisio is cross-platform, easier to integrate with other scientific software (often written in Java), and works with web technologies such as Java applets and Java Webstart. PathVisio already integrates well with some other Java-based scientific tools such as Cytoscape[3] for network analysis and Eu.Gene [4] for pathway statistics. Second, PathVisio uses a newly designed file format for storing pathway information that is extensible (XML-based) yet at the same time backwards compatible with the MAPP format used by GenMAPP. This means that the existing GenMAPP pathway archive can be used in PathVisio and other GPML-compliant programs. GPML has already been extended with new shapes and the capability to define relationships between nodes, allowing a network view of the pathway. Because of the novel features of GPML, it is preferable to use this format for pathway storage even if the MAPP format is used in later analysis steps. Third, the structure of the application is such that the pathway view and the data model are separated in different code modules. This enables the implementation of "copy," "paste," and "undo" commands, which are expected in modern user interfaces, yet are absent in GenMAPP. Separation of the data model also makes it easier to support different pathway file formats and image formats. PathVisio can be used to prepare illustrations suitable for publications with its vector graphics export feature. Finally, PathVisio re-implements GenMAPP's underlying gene databases with a new database schema that can be accessed more efficiently with fewer queries. These four technical improvements make the software more flexible, and open the possibility of new functionality and better integration with other tools.

The most important aspect of GenMAPP that has been mimicked in PathVisio is that the software places the biologist at the center. PathVisio works best in the hands of experimental biologists with a high level of domain knowledge. We aimed to avoid the situation where the software is so difficult to use that a specialized bioinformatician is needed. We want to gather pathway knowledge directly from the biologists who conceptualize pathways when designing and performing their experiments. This was our main guideline during the software design process. For example, we chose to use manual instead of automatic layout, to emulate presentation software that may be already familiar to the user. We chose locally installable synonym databases to make cross-referencing gene identifiers quick and automatic.

## Implementation

As noted above, the data model of PathVisio is completely separated from the rest of the application. It consists of two parts: the pathway data model and the synonym database model.

**Figure 1:**
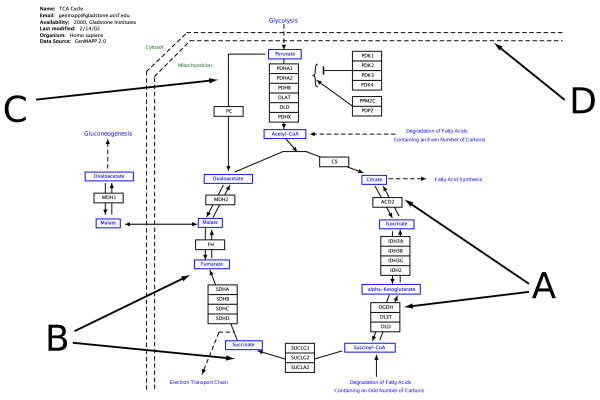
**Pathway data model**. A biological pathway as represented by PathVisio has three main classes of objects: *DataNodes*, *Lines *and *Shapes*. The most important are the DataNodes, represented by boxes. These data nodes can represent genes, proteins (A), or metabolites (B). DataNodes can be linked to an online database; in this example, MDH2 is linked to Entrez gene accession no 4191, and Malate is linked to HMDB identifier HDMB03256. DataNodes can be grouped to represent certain biological relationships. In this example, IDH3A, IDH3B and IDH3G are grouped to indicate that they form three subunits of a protein complex. A second class of objects is formed by lines, t-bars, and arrows that represent interactions between data nodes (C). Various shapes and text labels can be used to explain the pathway. In this example, shapes are used to distinguish the cytosol from the mitochondrion. (D). Pathways are stored in the GPML file format [see additional file 1] The PathVisio source code includes an XML Schema definition that can be used for checking the validity of GPML files.

In the pathway data model (Figure 1), there are three main types of objects in a pathway: *DataNode *objects represent biological entities, *Line *objects represent various types of interactions, and *Shape *objects serve as graphical annotation. The term DataNode is similar to the GenMAPP term GeneProduct, but we chose to use the former to show that it can be used to refer to any type of biological entity, not just genes and proteins. DataNodes can be grouped to show that they are biologically related (e.g., for paralogous genes or proteins in a complex).

To store this pathway model, we developed GPML or GenMAPP Pathway Markup Language. GPML is backwards compatible with the GenMAPP MAPP format, meaning that all information stored in MAPP format can be stored in GPML as well, and pathways can be readily converted. GPML has a set of extensions on top of the initial requirement of compatibility. It allows storing relations among different elements, so that a graph can be derived from a pathway. A pathway using this facility can be converted into a network, something that is impossible with the MAPP format. The utility of this feature will be discussed below. It also can link to BioPAX [5,6]. BioPAX is an emerging pathway standard for exchange of pathway data. The current version of BioPAX (level 2) lacks the ability to store coordinates and graphical annotations that are part of the GenMAPP format. By linking GPML elements to BioPAX elements, both are extended in XML fashion. In this way, GPML could be used as a presentation layer on top of BioPAX. To handle various pathway file formats, PathVisio makes use of a generic import/export interface.

The second part of the model used in PathVisio is the synonym database model. A variety of online genome databases are available to the bioinformatics community, leading to multiple identifiers for the same gene. One solution to this problem would have been to standardize on a specific database and let users make use of external services such as DAVID [7] to translate between ID types. This extra step for the user can be cumbersome and prone to errors. PathVisio uses another solution also implemented by GenMAPP, letting the software handle the translation through synonym databases.

**Figure 2:**
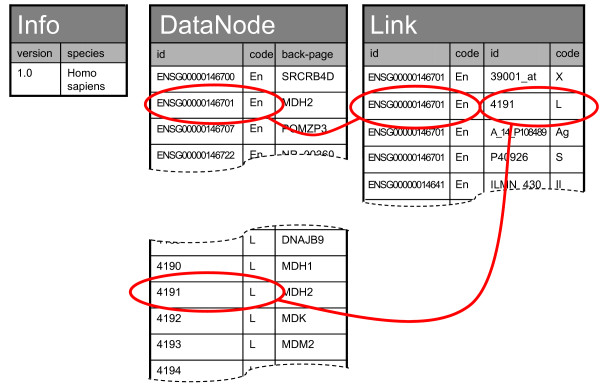
**Synonym database schema**. This figure represents the database schema for the synonym databases. There are three tables, Info, DataNode and Link. Info provides meta-data on the database. DataNode provides per-gene information, and Link provides a many-to-many relation between entries in the DataNode table that is used to store cross-references. The id column contains accession numbers from online biological databases. Systemcode is a short code (usually one or two letters) that represents the biological database that the id belongs to. A back-page is a short summary of the entity with HTML mark-up, containing at least a one-line description.

Synonym databases (called gene databases in GenMAPP) can be downloaded from the website pathvisio.org. Because this type of database is potentially used intensively, we chose to create locally installable versions rather than relying on a slow web-service. The synonym database schema (Figure 2) consists of three tables: "Info", "DataNode" and "Link". Info provides meta-data on the database. DataNode provides per-gene information, including a short description. Link provides a many-to-many relation between entries in the DataNode table that is used to store cross-references.

Synonym databases that we produce are based on Ensembl [8] and can in principle be made for any species that is annotated in that database. They are produced based on the Derby relational database system [9], because Derby can be packaged with the PathVisio software making installation easier. However, PathVisio is not tied to a specific database back-end. Depending on speed, usability and cost requirements, a different embedded or client-server database system could be used through the Java DataBase Connectivity (JDBC) layer. The use of synonym databases is not restricted to gene information; metabolites can be used as well to unify PubChem[10], ChEBI[11] and HMDB[12].

For viewing pathways, we chose an implementation based on the Java Graphics2D API, which makes it possible to output to screen as well as various file types. The flexibility of this API makes it trivial to add Shape types in the future. The Batik SVG Toolkit [13] is used for exporting to graphic formats, including those suitable for publication.

## Results and discussion

We envision three ways in which PathVisio could aid biologists doing research.

### 1: Organization of biological information

Many research questions are related to biological pathways in some way. For example, which receptor is responsible for carrying a stress signal across the nuclear membrane? Which proteins need to be activated to lead to a choice between two possible differentiated cell types? In these cases, a question is asked about a certain unknown component of a biological pathway. Experimental results could lead to a conclusion in terms of adding new elements to a pathway, clarifying the role of an element in a pathway, or proving the existence of an interaction between two elements. In all cases, biological knowledge is increased, and since pathways are a representation of this knowledge, the pathway itself is improved as a direct result of research. Expressing biological knowledge visually as a pathway can be a very powerful tool to organize disparate bits of information.

What is the best way to represent biological concepts graphically? There are certain conventions for this, as well as several published formalized symbolic languages [14-16]. The style used by GenMAPP and many textbook pathways does not pose many restrictions (e.g., an arrow can be used to mean stimulation, transport to a new compartment or simply interaction between proteins).

Molecular Interaction Maps (MIMs) [16] is one attempt to codify biological knowledge in schematics. The advantage of MIMs is that they store a large amount of information in a single diagram, and a knowledgeable person can retrieve all this information from the diagram with certainty.

**Figure 3:**
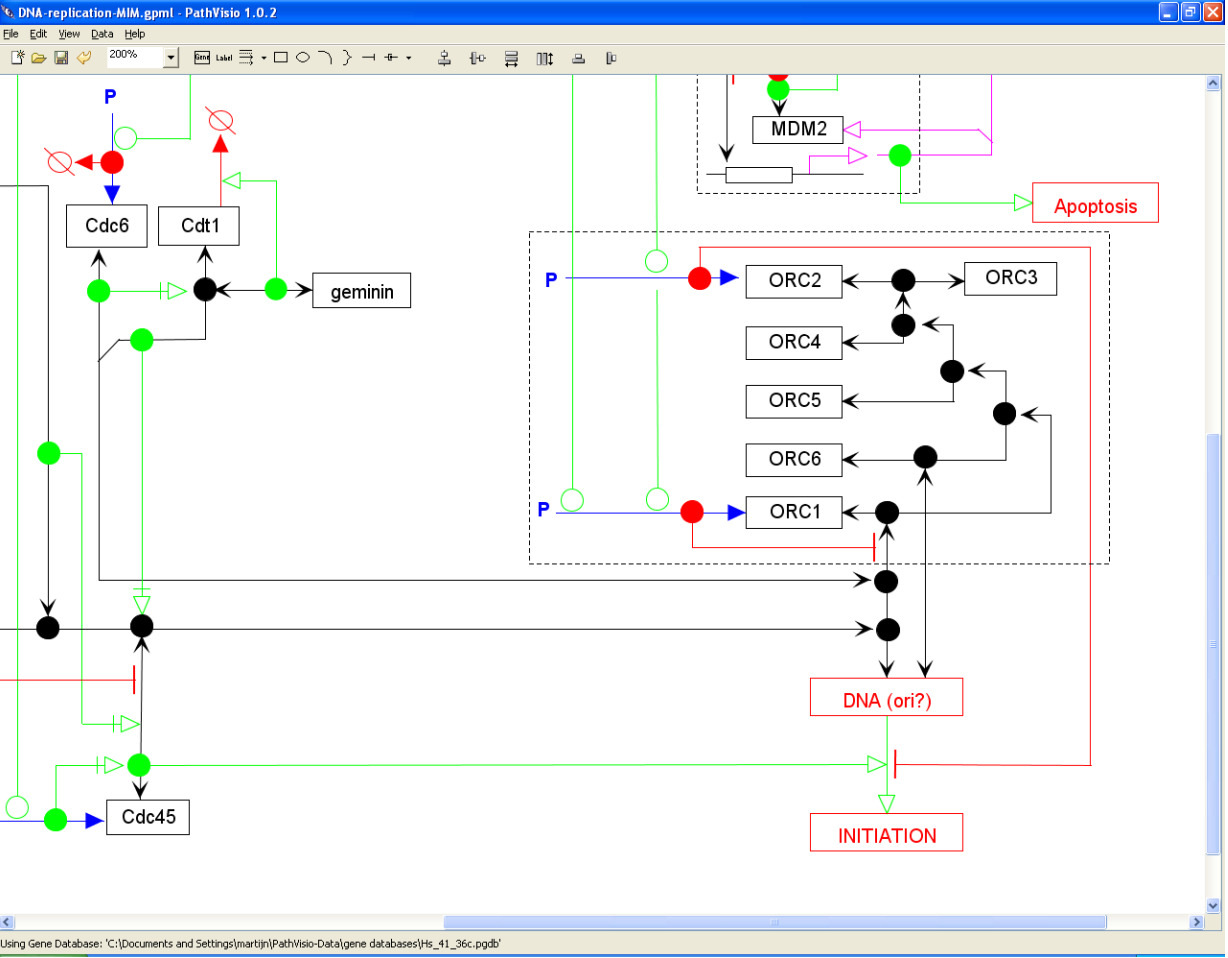
**Example of a Molecular Interaction Map drawn in PathVisio**. The original source for this pathway was published previously by Aladjem et al.[22] The pathway is stored in GPML format [see Additional file 2].

The current version of PathVisio (version 1.1) has some support for MIMs; many of the needed graphical elements can be drawn. Work is underway to make this support complete, including making complex aspects of MIMs, such as contingency arrows, available in a user-friendly manner (Figure 3). PathVisio is the first graphical editor with support for the MIM style. We hope other editors will support MIMs in the future, so that they will be established as a standard.

Kitano [17] proposed that a pathway schematic must be semantically and visually unambiguous. The requirement of unambiguously defining a pathway is necessary for computational simulation, but in our opinion this is simply impossible for most biological pathways. The complexity of these data can be confusing to the biologist or teacher who is attempting to convey fundamental aspects of the pathway. For example, the combinatorial explosion that arises when dealing with a protein that can be in different phosphorylation states can increase the complexity of the diagram. A trade-off exists between clarity and completeness. At the same time, the requirement for unambiguity hampers the iterative process by which a pathway can be compiled while research is ongoing. Many components of a pathway are unknown and unspecified as long as research has not yet provided a full mechanistic explanation of the subject. Kitano resolves this by proposing a reduced notation; similarly, Kohn [16] has added a distinction between heuristic and explicit maps. GPML allows ambiguity, and doesn't enforce a particular style of notation. By being flexible, GPML allows a pathway to progress seamlessly from a rough conceptualization to a well established pathway that can be modelled. As increasing levels of complexity are understood, any of the aforementioned styles can be used depending on the intended use of the pathway.

Biological knowledge stored in a collection of pathways is most useful if it is available online and frequently updated. An applet version of PathVisio is used for the WikiPathways [18,19] resource, where the research community can collaborate in the task of curating and updating pathway content.

### 2: Data analysis and pathway statistics

Data from high-throughput experiments such as microarrays can be combined with pathways to achieve new insights. Using pathways one can view data in its biological context rather than in an arbitrarily ordered table. Once a set of pathways have been collected in a repository, the possibility to do pathway statistics becomes available.

PathVisio supports this workflow in combination with the GenMAPP software suite. Pathways created with PathVisio can be exported to MAPP format. Subsequently, high-throughput experimental data can be loaded into GenMAPP, and user-defined color criteria established. DataNodes on pathways in GenMAPP can then be colored accordingly, putting relevant parts of the expression dataset together in a single view.

MAPPFinder [2], another tool in the GenMAPP software suite, can be used to search for pathways that are significantly regulated under experimental conditions. MAPPFinder counts how many genes on each pathway meet user-defined criteria and compares this to the expected number of genes that meet the criteria to calculate a z-score. These z-scores can then be used to rank a set of pathways, which is very useful in hypothesis-generating experiments to identify which biological processes are affected.

Eu.Gene can provide an alternative to MAPPFinder in circumstances where something other than z-scores is required. PathVisio can export pathways to the Eu.Gene gene list format. Eu.Gene can employ Gene Set Enrichment Analysis (GSEA) or Fisher exact tests for pathway statistics. We also assisted the Eu.Gene development team to allow direct import of GPML and visualization of pathways. These features will be available in the next Eu.Gene release

### 3: Network analysis and augmentation

Compilation of pathway information is necessarily a manual process, but it would be very useful to augment pathway information with computational tools, including text mining, data mining and interaction information from high-throughput datasets, such as yeast-two-hybrid data.

As an improvement on GenMAPP, GPML has been extended to store node-edge relations, making it possible to store true interactions. PathVisio allows the user to define interactions by joining two items with a connector line. Fore ease of use, the connector moves together with the element to which it is connected.

**Figure 4:**
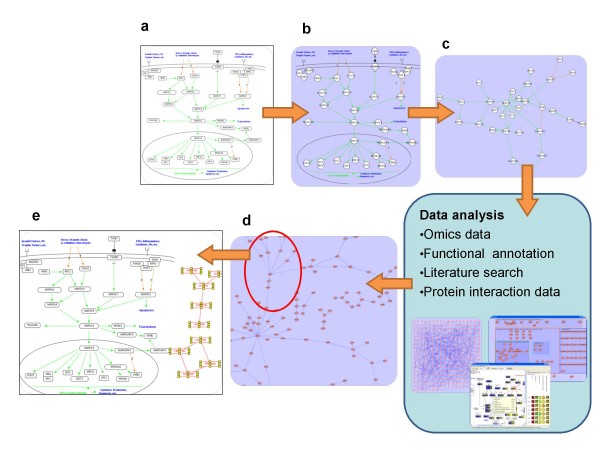
**Network analysis workflow**. This workflow suggests a way to combine manual pathway data and results from network analysis. A pathway in GPML format (a) can be copied to the Cytoscape network analysis program (b) with copy and paste commands. This program is useful for viewing the pathway as a network (c) without any extra graphical annotation. The rich plug-in set of Cytoscape can be used in various ways to extend this network. If the extended network contains new information that turns out to be instructive, it may be useful to transfer part of this network (d) back to PathVisio again (e) to add manual layout and graphical annotation.

The user-interface of PathVisio is optimized for the pathway model, meaning that PathVisio does not treat pathways as networks. The two concepts are closely related however, and network analysis of pathways can be useful. Other software, such as Cytoscape [3], is better suited to handle networks. Ideally, one would be able to use both programs together. Cytoscape supports a large set of plug-ins [20]. We created a GPML plug-in for Cytoscape that enables the user to transfer pathways between PathVisio and Cytoscape with copy and paste commands. This is the first step of a workflow for enhancing pathway information: 1, Create a pathway in PathVisio based on experimental results or literature research. 2, copy the pathway to Cytoscape. 3, Enhance the pathway using one of the many sources of interaction information available within Cytoscape. For example, the Agilent literature search plug-in could be used to obtain interactions from literature. The PathwayCommons plug-in makes various other sources of interaction information available. Once such interaction information is obtained, the network can be further enhanced, for example by grouping nodes by the cellular component they occur in using the BubbleRouter plug-in. 4, Copy the enhanced network back to PathVisio. 5, Re-arrange the network manually in PathVisio for presentation or publication. See figure 4 for a schematic overview of this workflow. All plug-ins mentioned in this workflow, including the GPML plug-in, can be downloaded directly using the Cytoscape plug-in manager.

### Future

For a specialized tool such as PathVisio to be relevant, it must be tightly integrated with other bioinformatics tools and standards. PathVisio can already be used in combination with Cytoscape through the GPML plug-in, and it is compatible with GenMAPP MAPP format and MAPPFinder for statistical analysis.

There currently exists a set of partly overlapping pathway format standards [6], and in our view, it is better to improve existing standards than to add new ones. As long as no pathway format completely solves all problems, the second best solution is to maximize compatibility and interoperability, and a move from a binary format to an XML-based format is a step in the right direction. GPML is intended to be a backwards compatible extension of the older, less flexible MAPP format. This step makes an older format easier to convert and more interoperable with other standards.

To clarify the role of GPML, we can compare it to other existing standards related to pathway definitions. BioPAX is a standard designed for exchanging data between pathway databases. GPML can embed elements from a BioPAX document and add visual annotations to it. This capability could facilitate integration of PathVisio with other pathway sources, such as Reactome, in the future. GPML is not suitable for computational modelling. Systems Biology Markup Language (SBML) is designed specifically for that purpose, and the overlap in scope of GPML and SBML is small. In the context of applications like CellDesigner [21], SBML defines reactions and parameters necessary for computational modelling. CellDesigner is a pathway drawing tool similar to PathVisio, but focused on creating graphical representations of SBML models. PathVisio and GPML instead emphasize flexibility and links to online databases, as these are valuable for human interpretation.

## Conclusion

PathVisio is fully open source, and GPML is an open format. We see open source as a necessity for this type of bioinformatics tool. Open source makes it possible for other tools to adapt to PathVisio and vice versa. With closed-source tools (e.g. CellDesigner and commercial packages), this adaptation can only go one way, which is a strong disincentive to collaborate. In a field where integration is of utmost importance, open-source software provides an optimal solution. To further encourage cooperative development, PathViso supports the addition of plug-ins, something that is facilitated by the Java programming language.

With PathVisio and GPML we have developed a framework for visual pathway analysis. This framework is very flexible with future extensions in mind. Development of PathVisio is ongoing. We wish to continue in the direction of increased flexibility and tighter integration with other bioinformatics standards and applications. This should ensure that pathway analysis and visualization can be done efficiently to improve biological research.

## Availability & requirements

Project Name: PathVisio

Project Home Page: 

Operating System: cross-platform. PathVisio has been tested on Windows XP, Ubuntu Linux 8.04 and Mac OS X 10.5.

Programming Language: Java

Other Requirements: Java 5 or higher

License: Free and open source under the Apache 2.0 License. There are no restrictions to use by non-academics. The source code is available at 

## Authors' contributions

All authors have read and agreed upon the content of this article. The PathVisio software was designed and written by MvI, TK, AP and KH. SC performed invaluable beta testing. AP and BC created the GPML concept, CE the PathVisio concept. MvI and SC drafted the paper.

## Supplementary Material

Additional file 1 **Krebs cycle pathway in GPML format**. Rendering of the Krebs cycle in GPML format, derived from the GenMAPP archive. This pathway is shown in figure 1. This file can be opened from within the PathVisio application. Click here for file

Additional file 2 **DNA replication pathway in MIM Style in GPML format**. Rendering of the DNA replication pathway using the MIM style, derived from Aladjem et al[22] Part of this pathway is shown in figure 3. This file can be opened from within the PathVisio application. Click here for file
